# Neural limits of temporal feature binding in human visual cortex

**DOI:** 10.1038/s41467-026-78974-z

**Published:** 2026-10-26

**Authors:** N Schönemann, C Merkel, H Strumpf, AM Felßberg, MV Bartsch, MA Schoenfeld, J-M Hopf

**Affiliations:** 1 Otto-von-Guericke University, Magdeburg, Germany; 2 Leibniz-institute for Neurobiology, Magdeburg, Germany; 3 Donders institute for Brain, Cognition and Behaviour, Radboud University, Nijmegen, The Netherlands; 4 Kliniken Schmieder, Heidelberg, Germany; 5 Center for Behavioral and Brain Sciences, Magdeburg, Germany

**Keywords:** Attention, Perception

## Abstract

Humans are able to track the change of color and orientation (CO) of an object at a stunning rate. However, spatially separating those features slows this rate by an order of magnitude – a temporal limit of feature binding that is little understood. Here, we investigate the underlying mechanisms using high-temporal resolution cortical source localization of the magnetic field response elicited by CO-pairs alternating at different frequencies in color and orientation. We show that (1) binding requires a rapid recurrent modulation (~120 ms) of activity in V1/V2, whose latency is independent of the alternation rate. (2) Performance drops to chance when the alternation rate becomes too high to accommodate the recurrent process within a single alternation. (3) CO binding itself is mediated by color-based attention, which gradually biases re-occurring CO combinations containing the attended color. These observations together demonstrate that CO-binding across space depends on rapid recurrent processing in early visual cortex, with its temporal limit being set by the time this process takes to be completed. Binding ultimately results from a slower and gradual attentional bias of the recurrent process instantiating a form of stroboscopic stationarity in the stream of alternations for unambiguous discrimination of the correct feature-combination.

## Abstract (web summary)

The speed of binding color and orientation in an alternating stream is limited by the duration of a recurrent process in visual cortex. Binding is accomplished by a gradual attentional bias of this process, causing a form of stroboscopic stationarity.

## Introduction

**Fig. 1:**
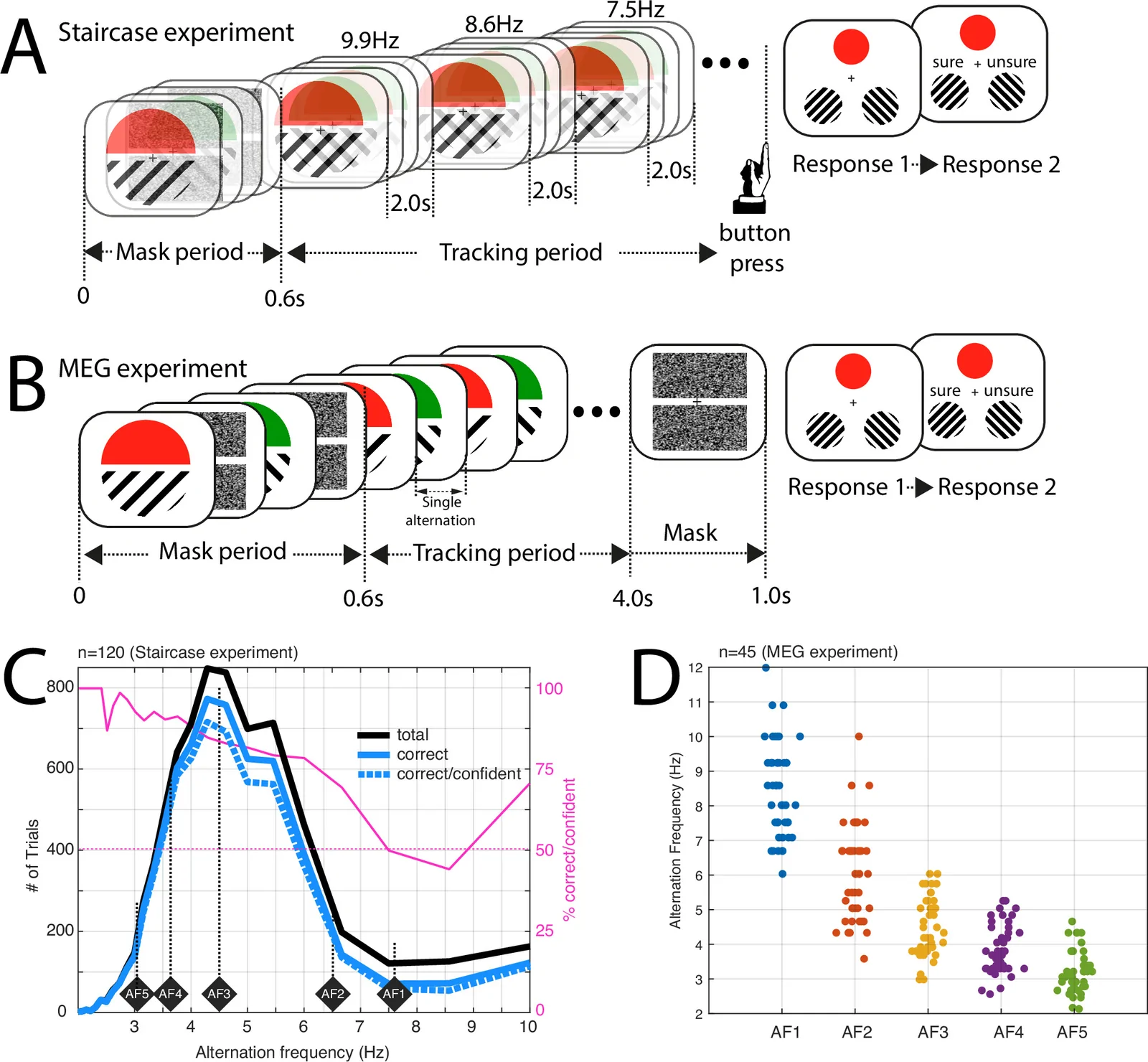
Experimental design and behavioral performance data. **A** Trial structure of the behavioral staircase experiment. **B** Trial structure of the MEG color-orientation binding experiment. **C** Number of trials (sum over *n* = 120 participants) at which participants reported a CO-combination as a function of the alternation frequency (AF). Black, blue solid, and blue dashed lines show the total number of responses, the number of correct, and correct confident responses, respectively. The percentage of correct confident responses of the total number of responses is shown in pink. **D** Plot of the alternation frequencies (AF1-AF5) of each individual observer (*n* = 45) obtained in the staircase experiment and used in the MEG binding experiment.

Modern humans live in dynamic visual environments with increasing demands on tracking simultaneous change. For example, when driving a car in dense traffic, we need to track the speed and direction changes of cars approaching us. When a brake-light suddenly turns on ahead of us, we must instantly realize what car it belongs to, and how fast we are approaching it. In such situations, critical features need to be properly combined in an instant of time to identify relevant objectse - a process referred to as temporal feature binding. It turns out that temporal binding of simple feature conjunctions like color and orientation can operate at a fast rate of ~19–35 Hz when the features occupy the same spatial location^1–4^, implying that such combinations are coded as integrated representations within the feedforward sweep of processing in the visual system. At such a fast rate, binding is assumed to be mediated by units in early visual cortex that code feature combinations, either by being tuned to combinations^5–12^, or by multiplexing temporal codes for different features^13,14^. However, a spatial separation of the color and orientation features slows the speed of selection by an order of magnitude, suggesting that binding across space is brought about by time-consuming neural operations^3,4,15^. Those are proposed to depend on feed-back processing in visual cortex^12,16–18^. To date, the cortical mechanisms underlying temporal binding of changing feature-combinations and their temporal limits are little understood. To explore those mechanisms in human visual cortex, we employ temporally resolved source localization of the visual evoked magnetic field response elicited by alternating color-orientation (CO) pairs as illustrated in Fig. 1A, B. The stimulation setup was constructed by following the experimental setup reported in Holcombe and Cavanagh^3^, where participants had to discriminate the correct combination of CO pairs alternating at different rates.

## Results

### The speed of color-orientation binding (Staircase experiment)

The staircase experiment was run before the MEG recording session to determine the individual range of alternation-frequencies (AF) where a given participant would be able to report a correct CO combination. To this end, participants (*n* = 120) were presented with CO patches alternating in descending frequencies starting at 10 Hz (Fig. 1A). Each AF remained on screen for 2 s and was then replaced by the next lower frequency (see Materials & Methods for details) until participants reported a feature pairing. Participants were also asked to indicate their confidence of choice with a second button press (confident, not confident). The results are summarized in Fig. 1C, which plots the total number of trials on which subjects were responding at a given AF (black), together with the number of correct (blue solid) and correct-confident responses (blue dashed). The proportion of correct-confident to the total responses is shown in pink. As visible, subjects responded most frequently in a range around 4–5 Hz where they were better than 75% correct. Responses started to become less frequent around ~6.5 Hz (AF2), with accuracy falling to chance level (50%) at ~7.5 Hz and higher. The lower limit of the response range appears around 3 Hz (AF5).

A subset of *n* = 45 participants was included in the MEG experiment, in which fixed AFs were used to investigate the cortical processes underlying temporal CO-binding. To this end, we defined five individual AFs (AF1-5) to cover the performance range of a given participant in the following way (see illustration in the overall histogram in Fig. 1C): We first defined the AFs at the upper (AF2) and lower limit of the response range (AF5) as indicated above. AF1 was then defined by incrementing AF2 by 2.5 Hz in order to enter a frequency range where a given subject would be unable to perform the task. AF3 was defined as the frequency where the participant gave the maximum number of responses. AF4 was defined as the median distance between AF5 and AF3. Figure 1D plots the alternation frequencies of all subjects participating in the MEG experiment.

### Cortical source activity elicited by alternating CO-pairs (MEG-binding experiment)

**Fig. 2:**
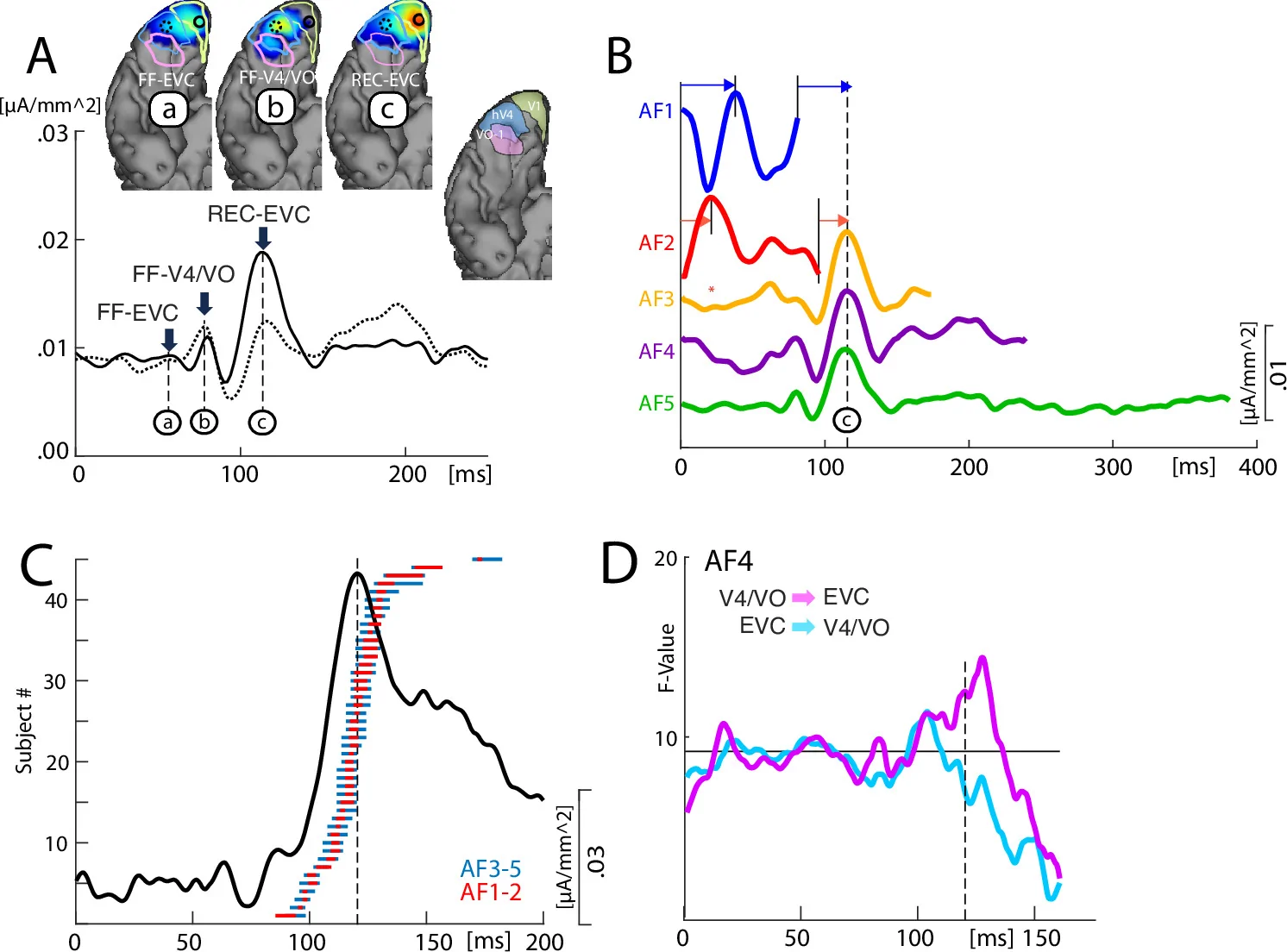
Time course and distribution of source activity in visual cortex. **A** Time course and topographical distributions of source activity in observer S1 elicited by the AF5 condition. Source waves represent average activity in ROIs in EVC (black solid trace and circle) and V4/VO (black dashed trace and circle). The topographical maps (a–c) show the cortical source distribution at three successive time points after alternation onset (dashed black vertical lines). Maps are set to an individual threshold showing activity above half of the source maximum at a given time point. The source maximum in (a) reflects the initial feedforward response in EVC (FF-EVC, a), which is followed by a maximum in mid/higher-level extrastriate areas (FF-V4/VO, b), which is in turn followed by a maximum re-appearing in EVC (REC-EVC, c). **B** Source waves elicited in the EVC-ROI as a function of AF1-5 in observer S1. The blue and red arrows on the left side measure the peak latency of the REC-EVC in AF1 and AF2, respectively. Adding those latencies to the AF (arrows at the right side) illustrates that the REC-EVC peak appears at a rigid latency that spills into the next alternation. **C** Latency range of the EVC-peak variation across AFs (horizontal bars showing shortest to longest latency) of the AF1-2 (red) and AF3-5 condition (blue) in each individual observer together with the average (*n* = 45) source wave in EVC of the AF4 condition (black solid). Note, the average was truncated at the latency exceeding the alternation time of the subject with the highest AF4. **D** Granger causality analysis (AF4 condition) testing the causation of activity in the V4/VO-ROI by activity in the EVC-ROI (cyan trace), and the causation of activity in the EVC-ROI by activity in the V4/VO-ROI (purple trace). The horizontal line indicates the statistical threshold-level (critical F-Value), above which the causation effects are significant. The vertical dashed line marks the latency of the peak amplitude of the average REC-EVC shown in (**C**).

To characterize the cortical processes elicited by the alternating stimulus-pairs, we averaged the brain response elicited by single alternations within an experimental trial, and then computed the average across all trials of the experiment in a given AF. Figure 2A shows the source waves elicited by AF5 trials in early visual cortex (EVC, encompassing V1 and V2, solid trace) and ventral extrastriate areas V4/VO (dashed trace) in one observer (S1). As visible, there is an initial small maximum around 60 ms (time point (a)) reflecting the initial stimulus-driven feedforward (FF) activation of early visual cortex areas (FF-EVC). Around ~85 ms (time point (b)), a source maximum appears in a region consistent with activity in mid-level/higher-level ventral extrastriate cortex V4 and VO1 (FF-V4/VO). This is followed by a maximum of recurrent activity in early visual cortex (REC-EVC) around 115 ms (time point (c)), consistent with a rapid feed-back (FB) modulation of EVC activity. Figure 2B shows the source waves elicited in EVC by the five AFs (AF1-AF5) in observer (S1). The waveforms are of different length due to the alternation time becoming shorter with increasing AFs. The source waves elicited by AF1 and AF2 are notably different from those elicited by AF3-5, as the large response peak indexing the response in EVC (blue and red arrows, REC-EVC) arises too early in the analysis interval to be elicited by the stimulus-driven FF response in EVC. This is particularly apparent for AF2, where the maximum appears 20 ms after alternation onset. The reason is that for AF1 and AF2 the alternation time (A-time) is too short to accommodate the FF-REC cycle such that the REC-EVC response spills over from the previous alternation period. This becomes obvious when adding the latency of the REC-EVC peak of AF1 and AF2 to the corresponding A-time as illustrated by the blue and red horizontal arrows in Fig. 2B. The latency of the REC-EVC peaks plus the corresponding AF-time appear almost exactly at the REC-EVC peaks of AF3-5, indicating that the timing of the initial FF-REC cycle is constant and does not change as a function of the AF. To validate the generality of this finding, we analyzed the peak latency of the REC-EVC in each individual observer. Figure 2C plots the range of peak-latency variation (range between shortest and longest latencies) for AFs 3-5 (blue horizontal bars) and for AFs 1-2 where the A-time was added to the REC-EVC (red horizontal bars) of each individual observer (*n* = 45). For orientation, the grand average REC-EVC across all observers of the AF4 condition is overlaid (black trace). Figure 2C shows that the REC-EVC peaks vary substantially among subjects, with latencies ranging from just under 100 ms up to ~150 ms after alternation onset. Relative to this ~60 ms variation, the mean temporal variation within subjects was very low (4.6 ± 3.5 ms). For testing the latency of the REC-EVC peak across all AFs (AF1-5), the alternation time (A-time) was added to the peak latency measure in AF conditions with the REC-EVC spilling-over into the next alternation. The analysis confirms that the peak latency does not vary as a function of AF (*F*(4,44) = 0.43, *p* = 0.785, *η*^2^ = 0.01, 90% CI [0.000, 0.106]), indicating that the REC-EVC appears at a subject-specific but a quasi-fixed latency that is independent of the AF.

Finally, the delayed source maximum in EVC (REC-EVC) relative to V4/VO (b to c in Fig. 2A) was taken to suggest that activity in EVC is rapidly modulated by FB from V4/VO. However, a delayed activity in one area relative to another is not sufficient to verify that the latter is modulated by FB from the former area. In fact, it is possible that the FF response in EVC is associated with a delayed maximum due to ongoing local processing independent of FB. One way to assess this possibility is to analyze the causal relation between the time-course of activity in EVC and V4/VO using a Granger causality analysis (GCA). Figure 2D shows the average (over participants) time-course of the GCA statistics (F-values) testing the feedforward causation of activity in V4/VO by EVC (cyan trace) and the feedback causation of EVC by V4/VO (purple trace) for the AF4 condition. Starting around ~110 ms, there is a significant increase of the feedback over the feedforward causation. The increase appears in the time-range of the REC-EVC and peaks slightly after the overall maximum of the REC-EVC (dashed vertical line) supporting the interpretation that FB from V4/VO contributes to the cortical response reflected by the REC-EVC.

### Binding performance is temporally limited by the time course of recurrent activity in EVC

**Fig. 3:**
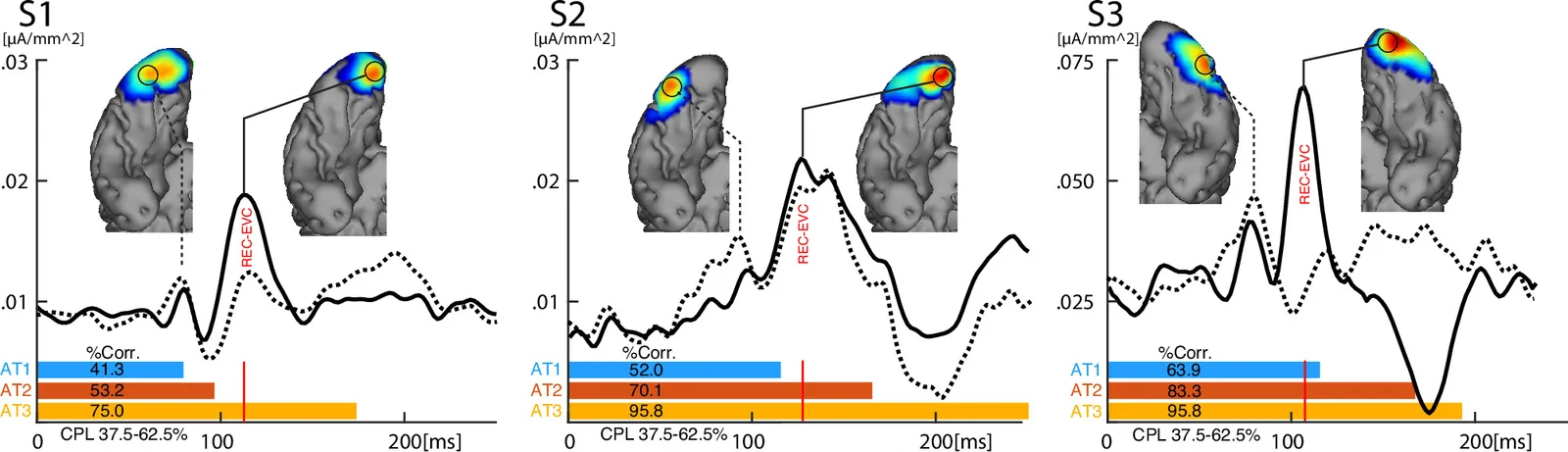
Source activity in EVC and V4/VO in relation to AF1-3 in three selected observers (S1-3). The horizontal color bars under the source waves plot the duration of the alternation time (AT1: blue, AT2: red, AT3: orange) in relation to the time course of source activity of the AF5 condition in EVC (solid trace) and V4/VO (dashed trace). The number in the colored bars indicates the percentage correct/confident responses (%Corr.) for the corresponding AT. The red vertical bar highlights the peak latency of the REC-EVC. Chance performance level (CPL) ranged from 62.5-37.5% correct responses.

Figure 3 illustrates the cortical response (AF5 condition) in EVC (solid trace) and ventral extrastriate areas V4/VO (dashed trace) in relation to the duration of AT1, AT2, and AT3 (blue, red, and orange horizontal bar) in three observers who are representative for the spectrum of performance variation across all participants. The individual performance level (% correct/confident responses, %Corr.) for a given AF is indicated by the number inside the bar. Chance performance level (CPL) ranged from 37.5–62.5%. The left panel (S1) displays the response of the observer already shown in Fig. 2, who showed chance performance at AF1 (41.3%) and AF2 (52.2%), but performed above chance at AF3 (75%). If we analyze the time course of source activity relative to the alternation time (A-time) in AF1-3 (highlighted as blue, red, and orange horizontal bar), we see that the A-time of AF3 contains the REC-EVC peak (peak latency of the REC-EVC indicated by red vertical line), while it is not contained in AF1 and AF2, suggesting that successful binding requires the REC-EVC response to be sufficiently contained in the A-time. This interpretation is consistent with the observer shown in the middle panel (S2), who performs at chance at AF1 (52%), but clearly above chance at AF2 (70.1%). Here, the REC-EVC is contained in the A-time of AF2, but not in that of AF1. Finally, the observer shown in the right panel (S3) shows above chance performance already at AF1 (63.9%). Consistently, a substantial portion of the REC-EVC response fits into the A-time of AF1. Note, based on the performance data of the staircase experiment, AF1 was defined to cause chance performance in all observers. Nonetheless, a few observers like S3 were able to perform above chance at AF1 during the MEG experiment. While we have no definitive explanation for the improvement, it likely results from a conservative response strategy and/or from training effects during the staircase experiment. To summarize, the performance pattern across the three observers suggests that the REC-EVC is mandatory for correct temporal binding across locations. That is, the cortical processes underlying the REC-EVC needs to be completed within the time of a single alternation, and before the feedforward sweep elicited by the following alternation compromises this process.

**Fig. 4:**
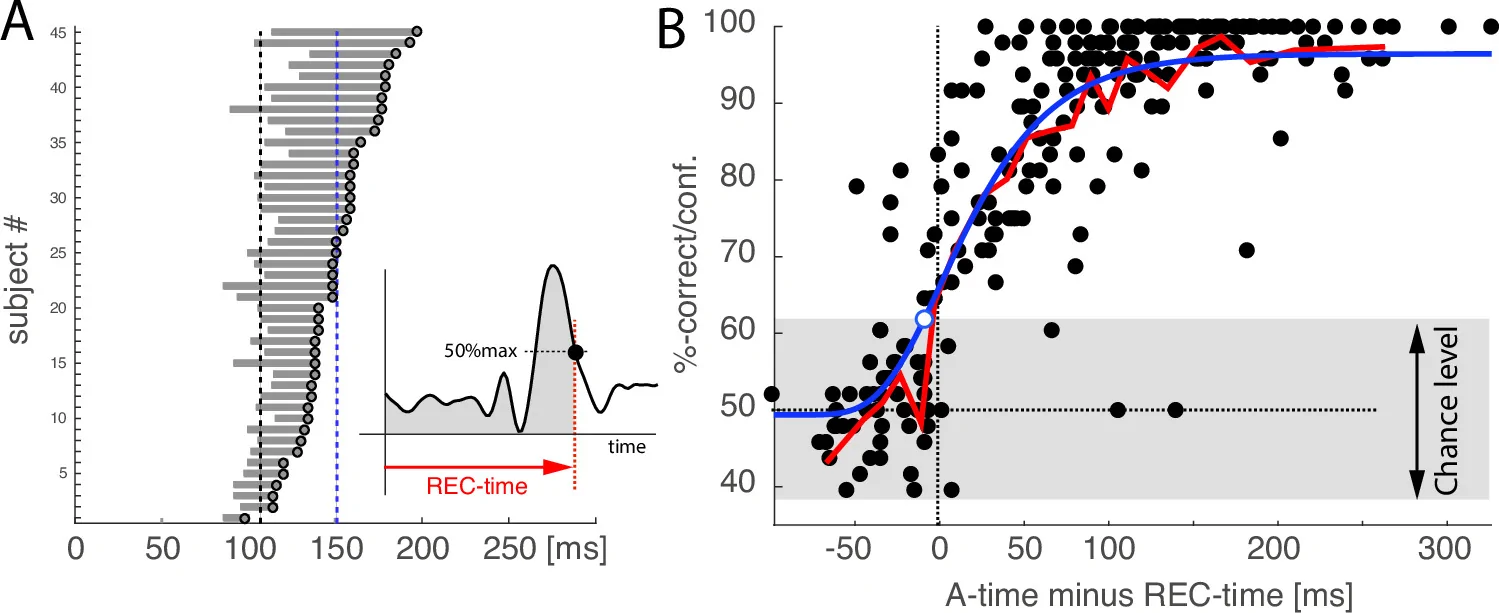
REC-EVC latency to performance relation. **A** Latency range of the REC-EVC response (grey horizontal bars) of each individual observer. The black circles mark the recurrence-time (REC-time), defined as latency at half-peak-maximum of the downslope of the response (see inset). The black and blue dashed vertical lines highlight the average (across subjects) begin (half-peak-maximum of the upslope) and end (REC-time) of the REC-EVC response. **B** Scatterplot of percent correct/confident responses as a function of the difference between the A-time and the REC-time of all AFs. The performance average within successive time-bins is plotted in red. The sigmoid function fit of the data is shown in blue. The blue circle marks the time point at which performance enters chance level.

To validate this interpretation, we determined the latency of the REC-EVC (REC-time) in each observer and AF condition, and then analyzed the binding success as a function of the temporal relation between the REC-time and the A-time. Figure 4A plots the time range of the REC-EVC of each individual observer sorted according to the latency of the REC-time. The range of the REC-EVC was measured as the range between the up-slope and down-

slope of the REC-EVC peak reaching half of the peak maximum amplitude. The REC-time was finally defined as the latency of the half maximum down-slope of the REC-EVC (black circle, see inset in Fig. 4A), with the reasoning that at this point the cortical processing underlying the REC-EVC will be sufficiently completed. Figure 4B plots the binding performance (% correct/confident) against the time difference between the REC-time and the A-time, such that an increasing negative difference will indicate that the A-time was progressively too short to contain the REC-EVC. As visible, with the REC-time approaching the A-time, the performance declines gradually, but shows a clear drop into the range of chance performance (62.5–37.5%, grey zone, see Methods for details) when the A-time becomes shorter than the REC-time. This pattern becomes clear when plotting the performance as average within successive time-bins of 10 ms (red trace). The performance drops visibly to chance in the first time-bin where the REC-time exceeds the A-time. For further validation we fitted the data with a sigmoidal (Gompertz) function (see Methods for details), which yielded a high-quality fit (R-square = 0.747). Critically, the fitted curve (blue trace in Fig. 4B) enters the chance-performance range (blue circle) very close to the 0 ms mark (dotted vertical line) at −6.9 ms, confirming that performance reaches chance level when the A-time becomes shorter than the REC-time.

### CO-binding is associated with stronger recurrent activity in EVC (control experiment)

**Fig. 5:**
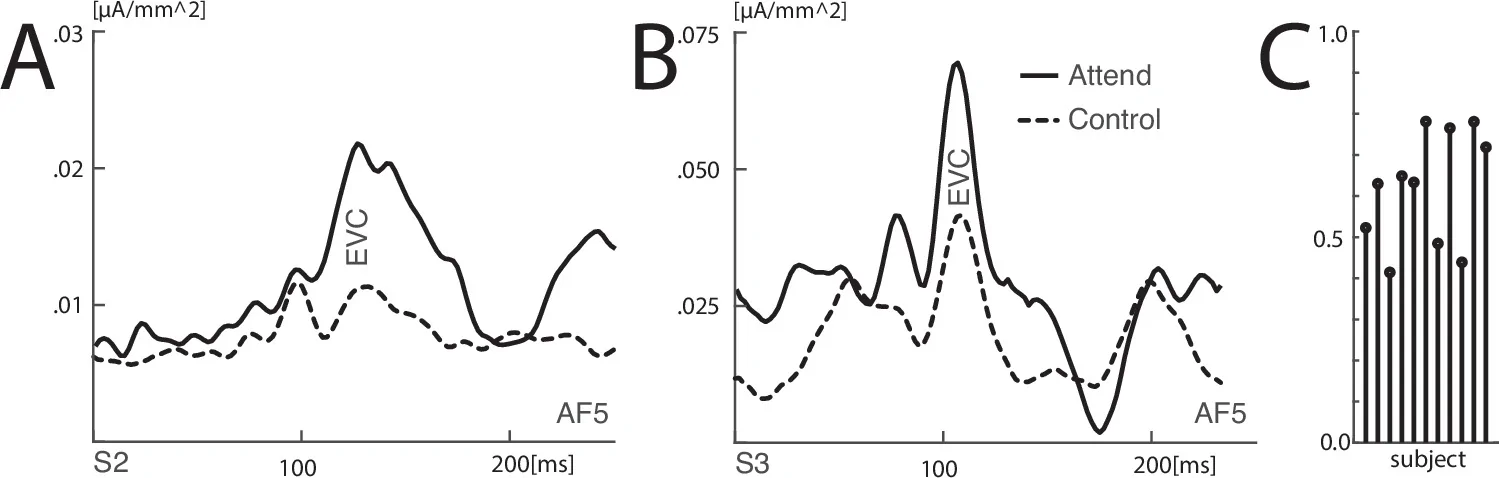
Results of the control experiment. **A**/**B** EVC source waves of control experiment (dashed) and the CO-binding experiment (AF5 condition, solid) in observers S2 and S3. The source waves of the control experiment were derived from the EVC-ROIs of the CO-binding experiment. **C** Amplitude of EVC-REC peak of the control experiment (AF5 condition) relative to the peak EVC-REC response of the CO-binding experiment (set to 1.0) in the *n* = 11 participants of the control experiment.

The analyses above suggest that the early REC-EVC is important for correct temporal binding across locations—an observation that dovetails with a previous demonstration that rapid recurrent processing (around 110 ms) in primary visual cortex is critical for target detection in an object-substitution masking task^19^, with successful target detection being associated with an amplitude enhancement of this early recurrent process. For the present data, we therefore predict that when the rapid REC-EVC is important for CO-binding, it should be larger relative to an experimental situation in which subjects are presented with the CO-alternations but do not perform the binding task. We tested this prediction in a subset of *n* = 11 participants of the CO-binding experiment. The CO-pairs were presented at the individual AFs used in the CO-binding experiment, but subjects were asked to ignore the stimulus alternation and detect rare occasions of a dimmer fixation cross, which appeared temporally random and unrelated to the AFs. Figure 5A, B illustrates the EVC response of the control (dashed traces) and CO-binding experiment (AF5 condition) in observers (S2 and S3). We clearly see an EVC response in the control experiment that peaks at the REC-EVC maximum of the CO-binding experiment. The REC-EVC amplitude, however, is smaller in the control versus the CO-binding experiment. Figure 5C plots the REC-EVC peak amplitudes of the control experiment as proportion of the peak amplitude of the CO-binding experiment in all participants of the control experiment. The response amplitude is on average 60% of that of the CO-binding experiment (*F*(1,10) = 28.15, *p* = 0.00034, *η*^2^ = 0.74, 90%CI [0.294, 0.851]), indicating that the CO-binding is associated with an enhanced REC-EVC response. This suggests that a modulation of the REC-EVC activity is a critical neural process required for CO-binding. It should be noted that the control experiment involves a change of the experimental task relative to the CO-binding experiment, with the potential that non-specific factors like differences in task difficulty could account for the overall smaller REC-EVC amplitude of the control experiment. However, performance was very high for both the AF5 condition of the CO-binding experiment (97.5%) and the control experiment (98.6%). A statistical comparison yielded no difference (*F*(1,10) = 1.15, *p* = 0.30, *η*^2^ = 0.1, 90% CI [0.000, 0.438]), rendering it unlikely that task difficulty was a major factor causing the larger REC-EVC amplitude in the CO-binding experiment.

### Recurrent activity in EVC builds up over the time-course of alternations

**Fig. 6:**
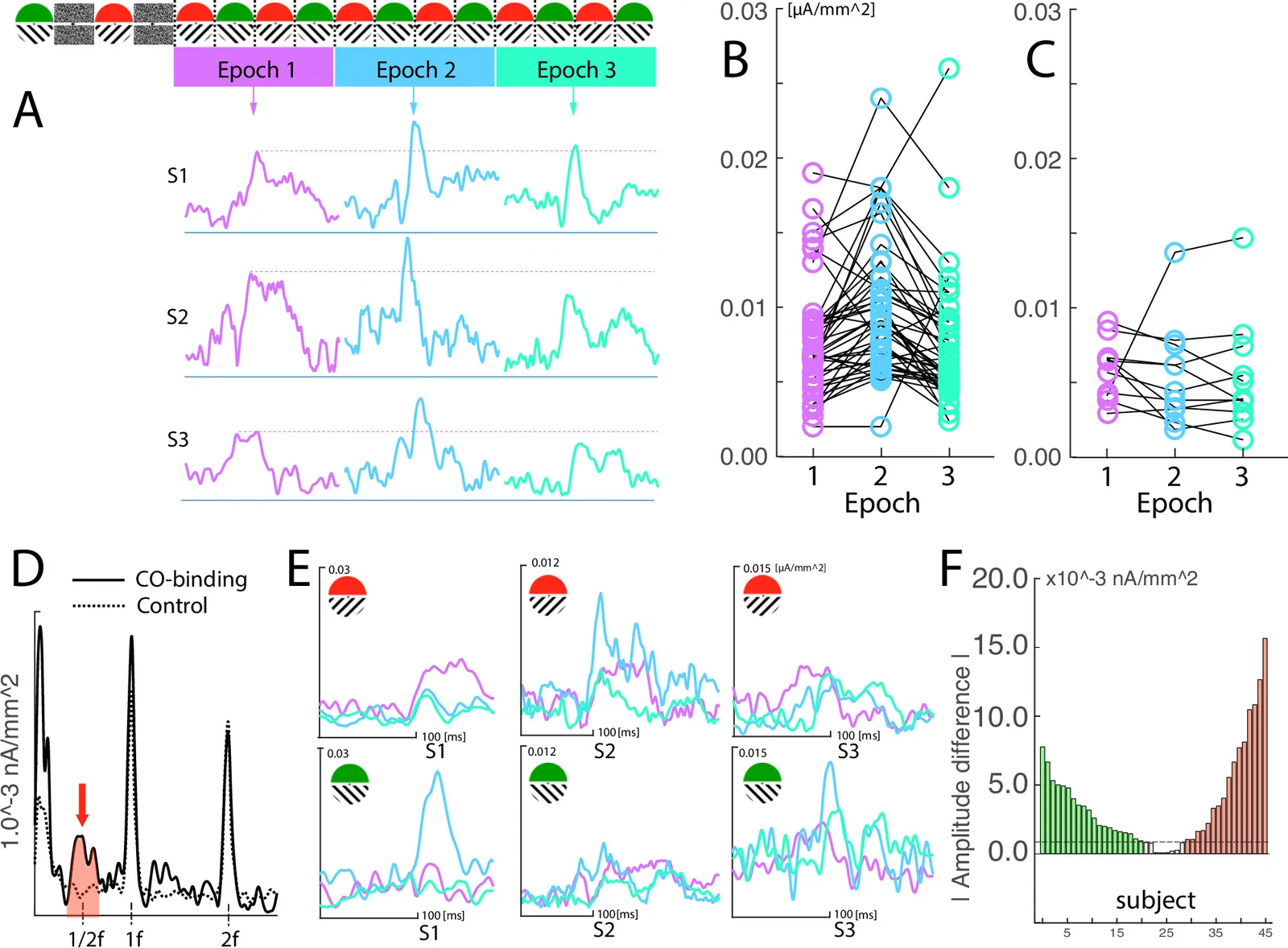
Epoch- and color-selective analysis. **A** EVC source waves of the CO-binding experiment in three subsequent epochs (containing for alternations each) of the tracking period (purple, blue, green) in observers S1-S3. **B** Individual amplitudes (*n* = 45) of EVC-REC peak of the CO-binding experiment in three subsequent epochs of the tracking period. **C** Individual amplitudes (*n* = 11) of EVC-REC peak of the control experiment in three subsequent epochs of the tracking period. **D** Spectral analysis of the average steady-state visual evoked response elicited in the tracking period (0.6–4.0 s) of the CO-binding experiment (solid) and the control experiment (dotted). The red arrow and area under the curve highlight the maximum at half the fundamental alternation frequency (1/2 f). **E** EVC source waves of the CO-binding experiment in three successive epochs, separately analyzed for CO-pairs containing red (upper row) or green (lower row). **F** Amplitude difference between EVC peak amplitudes for red minus green of each individual subject. The plot shows rectified differences with green larger than red responses (green) being plotted upward. The dashed horizontal line marks the confidence-level above which the amplitude differences are significant (Student’s *t* at *p* < 0.05, two-sided).

The control experiment shows that overall CO-binding is associated with an enhanced early REC-EVC response, suggesting that the operation of binding depends on a rapid top-down bias of the REC-EVC. This is consistent with the proposal that feature binding requires attention and feedback^16,20^ and adds to the observation that attention operates via feedback to EVC in other domains like spatial selection^21–24^. Notably, research using event-related potentials (ERPs) has shown that the ERP modulation reflecting sustained spatial focusing over occipital recording sites takes a few stimulus presentations to arise^25^, suggesting that attention establishes its modulatory effects in early visual cortex only gradually. An important question to investigate is whether the binding-related REC-EVC would show a comparable temporal dynamic. To address this question, we analyzed the peak amplitude of the REC-EVC as a function of its latency relative to the onset of the unmasked CO-pair alternations within an experimental trial. Specifically, we averaged the REC-EVC peak amplitude in consecutive epochs of an experimental trial (AF4 condition), with each epoch containing four successive alternations as illustrated in Fig. 6A. The figure shows the REC-EVC response for three consecutive epochs in the three example subjects shown in Fig. 2. In all three subjects the REC-EVC of the response of the second epoch (blue) displays a larger amplitude and a narrower peak than that of the first epoch (purple). In the third epoch (green), the narrow peak remains but shows a smaller amplitude than the second epoch. Figure 6B plots the REC-EVC peak amplitude of each observer and epoch, which verifies the pattern in the subject population. With only a few exceptions, the amplitude of the REC-EVC is significantly larger in epoch 2 than epoch 1 and 3 (*F*(2,44) = 11.08, *p* = 0.00005, *η*^2^ = 0.08, 90% CI:[0.104, 0.495]). An analogous analysis of the control experiment (Fig. 6C) shows a very different pattern. Except for one subject, the amplitude becomes slightly smaller with increasing epoch latency. The gradual decline, however, is not significant (*F*(2,10) = 0.15, *p* = 0.86, *η*^2^ = 0.01, 90% CI [0.000, 0.223]). In sum, the analysis of subsequent epochs indicates that the attention-driven REC-EVC enhancement builds up during repetitions of the stimulus towards a maximum roughly in the middle of the tracking period and declines again towards the end of the tracking period. The decline in epoch 3 relative to epoch 2 is likely to reflect the fact that on average, binding was attained during epoch 2, causing subjects to discontinue the attentional process of biasing the REC-EVC in epoch 3.

### Feature-based attention enhances recurrent activity in EVC

What biases the REC-EVC? There are at least two possibilities. One is that subjects use feature-based attention to bias one color (or orientation). This would enhance alternations containing this color (orientation), which in turn facilitates the readout of the associated orientation (color). Another possibility is that subjects employ rhythmic temporal attention^26^, which emphasizes every second alternation where the same CO combination reappears, thereby facilitating the readout of the same repeating combination. Given that subjects were always required to report the orientation paired with red, attending color would be the most likely strategy subject used to perform the task. Note, both mechanisms would cause a rhythmic bias of every second alternation. A spectral analysis of the steady-state visual evoked response in the tracking period should therefore show a response peak at half the alternation frequency (1/2AF). Figure 6D shows that this is indeed the case. At 1/2AF there is a clear enhancement (solid black trace, red arrow) that is not present in the control experiment, where subjects are to ignore the CO-alternations (dashed trace). Note, the 1/2AF enhancement does not reflect a lower harmonic of the fundamental stimulation frequency, as such harmonics do not appear below 10 Hz stimulation in the SSVEP response^27^. The 1/2AF enhancement is therefore an active enhancement due to participants allocating attention to every second alternation. In a further step, we asked whether feature-based attention causes the rhythmic bias of the REC-EVC response. To this end we averaged the REC-EVC peak amplitude separately for CO-pairs containing green versus red in consecutive epochs analogous to the analysis shown in Fig. 6A. As shown in example subjects S1-S3 in Fig. 6E, the REC-EVC amplitude enhancement is primarily seen for one color, with subjects S1 and S3 preferentially biasing green while S2 shows a bias for red. Figure 6F summarizes this color bias across all observers by plotting the rectified peak amplitude difference between red and green trials of the AF4 condition (average across Epochs 1-3). Subjects showing a green bias appear on the left side (green bars). The dashed horizontal line marks the confidence level (Student’s-*t* at *p* < 0.05, two-sided) above which the difference is significantly larger than zero. As visible, roughly half of the participants perform the task by biasing red and the other half by biasing green, with only 8 participants showing no bias towards one or the other color (white bars). Note, subjects could alternatively rely on a bias for a certain orientation but not color to facilitate the discrimination of the associated color. In this case, the red-versus-green analysis should show no difference between colors, because across trials the orientation was randomly paired with a given color.

## Discussion

Here we use MEG-based source localization to investigate cortical processes underlying the binding of alternating color-orientation pairs in human visual cortex. Testing *n* = 120 participants in a behavioral staircase experiment revealed that subjects start to be able to report the correct combination around 6.5 Hz, with the maximum number of correct/confident reports being given around ~4–5 Hz. This comparably low rate is well in line with previous observations reported in a similar experimental setting^3,4^. The analysis of the event-related neuromagnetic brain response revealed that each alternation of the stimulus sequence elicits a rapid cycle of feedforward from EVC (V1/2) to V4/VO followed by recurrent activity in EVC (V1/2). On average, the peak of this REC-EVC appeared at ~120 ms, and was completed around ~150 ms after alternation onset. The cycle time corresponds to 6.7 Hz, which is overall in line with the results of the staircase experiment (*n* = 120) where temporal binding of color and orientation started to raise above chance between 7 and 6 Hz (Fig. 2A). The latency of REC-EVC turns out to be an individual constant that doesn’t vary as a function of the AF, such that it spills rigidly into the next alternation period when the alternation time (A-time) becomes shorter than the latency of the REC-EVC (cf. Fig. 3A). Importantly, we find that performance falls to chance exactly at the A-time where the REC-EVC is not sufficiently contained. This indicates that the temporal integrity of the rapid recurrent process in EVC is a critical precondition of successful binding across locations. A control experiment reveals that the amplitude of the REC-EVC is larger when subjects perform the CO-binding task versus when they ignore the alternating CO-pairs, indicating that CO-binding is associated with a gain enhancement of rapid recurrent activity in early visual cortex.

### Feedback latency sets the temporal limit of CO-binding in visual cortex

The present experiments show that the modulation and temporal integrity of early recurrent activity in EVC is critical for successful CO-binding across space. The REC-EVC may reflect a rapid feedback of activity from higher-level area VO, aligning with the proposal that feature-binding requires feedback or reentrant processing from higher level-areas^12,16,17,28–31^. Here, this initial feedback appears rapidly around 100–150 ms after stimulus onset, which is overall in line with the onset of feed-back driven figure-ground segmentation processes in monkey V1^32,33^. Also, ERP data on figure-ground segmentation suggest early reentrant processing in human EVC to appear around 109-141 ms—a temporal range first disrupted by backward masking^34^. Converging observations were made with cortical inactivation of EVC via TMS, by showing that stimulus awareness is typically disrupted in two time ranges, with a late one reflecting feed-back processing between 80 and 140 ms^35–37^. Finally, the REC-EVC is consistent with previous MEG recordings documenting the canonical recurrence of V1 activity around 130 ms after stimulus onset in an object-substitution masking task^19^. The modulation of recurrent activity (110 ms) in Boehler et al., however, was found to mediate stimulus awareness, while attention appeared as later modulation in V1 (beyond 200 ms), suggesting a temporal dissociation of recurrent processes for awareness and attention^19,38–40^. There are substantial arguments for a dissociation of awareness and attention^41,42^. Note, modulations of cortical activity^43^ by feature attention and single unit responses in the monkey typically appear beyond 150 ms after stimulation onset^44^. Cortical feed-back underlying feature and spatial attention in human visual cortex is seen to arise around 180–200 ms after stimulus onset^21,22,45–47^. Hence, the temporal integrity of the REC-EVC seen between 100 and 150 ms appears to be a precondition of awareness on binding, which is subject to attention-driven top-down modulations.

### Attentional top-down bias of the REC-EVC builds up over time

Analysis of the REC-EVC peak revealed an amplitude increase over the course of the stimulus alternation, implying that CO-binding occurs as result of temporal integration over repeated alternations (see for an analogous account of the ultrafast color-orientation binding within an object^31^). The temporal integration would allow the slower feature-based modulations to take effect and bias the representation of a selected feature or feature-combination (see discussion below). Under this analysis, the observation that the REC-EVC must fit into the A-time represents a prerequisite for top-down attention to operate efficiently, i.e., on a stable and unambiguous EVC representation. This interpretation aligns with the proposal that EVC serves as a visual scratch pad or high-resolution buffer for integrating computations or testing predictions formed in higher level cortex^33,48–54^. This may even involve repeated sampling from EVC^55,56^. The attentional bias of the early feedback would gradually enhance the SNR of the scratch-pad representation such that at some point the feature pairing can be discerned. As already outlined, there are at least two mechanisms to realize this. Attention has been shown to operate in the time domain by biasing visual sensory processing following the temporal structure of stimulation^26,57–60^. One mechanism would therefore be to allocate rhythmic temporal attention to every second alternation on which the same CO combination reappears. Another mechanism would be to allocate attention to one color (or one orientation), which would then bias every second alternation on which the attended color (orientation) appears in combination with the same orientation (color). Color-based attention is the more likely mechanisms given that subjects had to report the pairing of the orientation feature with a fixed color. A separate analysis of red and green CO-combinations revealed that most participants, indeed, show a significantly larger REC-EVC amplitude for one over the other color, suggesting that a color-based attention is employed to aid binding by stabilizing the percept for discrimination over the course of a given trial. In this sense, attention mediates binding not so much by “gluing” features^31^, but by biasing an intact and unambiguous scratch-pad representation of one feature combination via a form of “stroboscopic” stationarity. Note, while the present experiments suggest that color-based attention aids binding in a stream of alternating CO-pairs via “stroboscopic” stationarity, they do not prove that this mechanism mediates binding in experimental situations where the pairing of features does not alternate but vary randomly, or where color attention would have to bias multiple color values in parallel. Future research will have to clarify whether feature-attention would mediate binding in a similar way under other experimental conditions. Finally, only a few subjects did not show any color bias, consistent with them employing rhythmic temporal attention.

### How is color and orientation information bound across spatial locations?

The present observations suggest that CO-binding is accomplished by color-based attention, which serves to bias the EVC scratch-pad representation towards an unambiguous percept akin to stroboscopic stationarity. The REC-EVC process may not reflect the operation of binding per se. Instead, if it remains uncompromised, it provides a stable input representation in which top-down attention can bias the read-out of feature pairings. In fact, an unaltered REC-EVC process may not only be a precondition for successful feature binding across space as investigated here, but also for other visual perceptual selection processes. In particular, it is possible that temporal limits set by the latency of the REC-EVC process may play a role in backward and object-substitution masking^19^. Future experiments will have to address the generality of this process. Furthermore, it is important to emphasize that the CO-binding assessed here does not refer to the process of linking different features of an object, but to the pairing of color and orientation values across locations, for which object-based mechanisms of feature binding^61^ may play no role.

How are color and orientation then linked across space given a temporally stable EVC representation? Several mechanisms are thinkable. Based on intracranial recordings in monkeys, Shipp et al.^12^ propose that units tuned to feature combinations in feed-back layers of early visual cortex areas bridge the top-down bias from higher-level units specialized for the selection of different feature dimensions, such that the biased output of feed-back layer units coding a specific combination realize the binding. Importantly, this mechanism could bridge the bias coming from separate areas representing different retinotopic locations in the visual system^49^. Another way to accomplish CO-binding across locations would be that color-units with large receptive fields in higher-level areas (encompassing both the color and orientation stimulus) impose a targeted top-down bias onto orientation-selective units in lower-tier areas on alternations where the biased color is presented. The CO-pairing would then be signaled by orientation-selective units showing the largest response under the color bias. Note, a similar proposal was made for a conjunction identification mechanisms via targeted feedback from orientation selective neurons by Vigano and colleagues^62^.

## Methods

### Participants

A total of 120 (mean age 37 years, 73 females) participants took part in the behavioral staircase experiment. A subset of 45 participants (mean age 33.4 years, 30 females) participated in the MEG CO-binding experiment. Eleven (mean age: 34.8 years, 7 females) participants of the binding experiment performed an additional MEG control experiment. Sex was determined based on self-report, but was not considered in the study design. All participants were right-handed and reported having normal neurological status and normal or corrected-to-normal (color-) vision. The recruitment of participants was conducted via the shared participant acquisition platform (SONA) of the Otto-von-Guericke University Magdeburg (OvGU), the Leibniz Institute of Neurobiology (LIN) Magdeburg and the DZNE Magdeburg. All participants gave informed agreement and received either financial compensation (10€/hour) or study credits (1/hour) for participation. The study was conducted according to the research regulations of the Declaration of Helsinki and was approved by the ethics board of the OvGU (study approval number: 54321). The data generated in this study have been deposited in the OSF database under accession code https://doi.org/10.17605/OSF.IO/6T325

### Stimuli

Stimuli were constructed in following the description in Holcombe and Cavanagh^3^ as close as technically possible. As shown in Fig. 1A, B, stimuli were pairings of spatially separated color-orientation (CO) half-circles, which were arranged as a horizontally split circle (diameter 9.9° of visual angle) with a horizontal gap of 0.4° of visual angle. The split-circle was centered onto a white fixation cross in the center of the back-projection screen. Each pair of half-circles consisted of a uniformly colored (red vs. green) upper half and a black-white sine-wave grating (tilted 45° either to the left or to the right) in the lower half. Stimuli were presented on a dark gray background (~8 cd/m2). The red and green half circles were set to equal luminance (approximately 17 cd/m2) as photometrically verified with a color spectroradiometer (CRS SpectroCal, Cambridge Research System).

### Apparatus

Stimuli were constructed and presented using Psychtoolbox 3 (Brainard, 1997; Pelli, 1997; Kleiner, Brainard & Pelly, 2007) in MATLAB R2021b and R2018a (Mathworks, Natick MA, USA), and back-projected onto a semi-transparent screen using Propixx (Vpixx Technologies Inc., Saint-Bruno, QC, Canada; resolution: 1920 × 1980; refresh rate: 120 Hz). Viewing distance was 1.0 m from the screen to the participant’s eye. The initial staircase experiment was conducted outside the MEG recording facility at a behavioral test laboratory using a 24-inch monitor (ASUS VG24SQE-3D; refresh rate: 60 Hz). The MEG was recorded inside a µ-metal shielded recording chamber (Vacuumschmelze, Hanau, Germany) using an Elekta Neuromag® TRIUX^TM^ MEG scanner with 102 sensor triplets, each containing one magnetometer and 2 planar gradiometer coils. Recordings were performed at a sampling rate of 1000 Hz and band-pass filtered between 0.01 and 300 Hz. For co-registration of the MEG data with the anatomical data of each participant, individual anatomical landmarks and 5 head position indicator coils were digitized in 3D space using the Fastrak system (Polhemus Inc., Colchester, VT, USA). Respective coils were placed on standard locations of the EEG-cap to track the participant’s head position during the recording. Head movements during the MEG-recordings were controlled for by measuring the signal of the position indicator coils before the first and after the last experimental block as well as during short pauses between blocks. Head movements were finally corrected relative to the last measured head position.

### Procedure

#### Staircase experiment

As illustrated in Fig. 1A, on a given trial the color-orientation pairs were presented with decreasing alternation frequencies (AFs), starting at 10 Hz and alternating with a mask stimulus (salt-and-pepper-noise-mask, noise-density: 0.5) for the first 0.6 seconds. Thereafter, the mask disappeared, and the CO-pairs alternated directly with each other, while the alternation rate stepped down every 2 s by incrementally adding one refreshment frame (16.67 ms). Participants were required to continuously attend the alternation stream until a correct discrimination of the CO-pairs was possible. Participants were instructed to respond correctly instead of quickly. The tracking-phase continued until the first button press. This was followed by a response screen requiring observers to indicate the color-orientation assignment (response 1). Specifically, the response screen contained one color patch and two orientation patches below. Subjects had to the select the orientation paired with this color via mouse (clicking the left mouse button with the index finger of the right hand). If no response was given, the trial timed out at an alternation rate of 2 Hz. After response 1, a further screen appeared where participants were inquired about the confidence (sure/unsure) of their response (response 2). Each participant performed a total of 60 trials.

### MEG CO-binding experiment

During the MEG experiment, the stimuli were presented at fixed AFs during a tracking-period of 4 s (trial). Participants initiated each trial via button-press. They were instructed to fixate the center cross until the response-screen appeared, and to avoid eye blinks during the tracking-period. As illustrated in Fig. 1B, the stimulation started with the CO-pairs alternating with a mask for 0.6 s (mask-period), was followed by a 3.4 s period of active tracking the alternating CO-pairs (tracking period), and ended with a mask presented for 1 s. Pilot experiments revealed that without masking, subjects would sometimes be able to discern the correct color orientation pairing on the very first and last stimulus frame even on very high AFs. To exclude this possibility, the stimulus alternation was masked at beginning and end of the tracking period. Participants were to report the orientation associated with the red color by pressing the index- or middle finger to select the left or right grating orientation, respectively. Subsequently, “confident” or “not confident” responses were to be given with the index- or middle-finger, respectively. Short pauses were placed in between blocks. Stimuli were presented in 4 separate blocks of 60 trials each. Within each block, the five AFs were presented in randomized order, 12 times each, resulting in a total of 48 trials per AF condition.

### Definition of alternation frequencies for the MEG experiments

Five individual AFs (AF1-5) were defined to cover the performance range of a given participant as illustrated in Fig. 1C. First, AFs were placed at the upper (AF2) and lower limit of the response range (AF5), i.e., the points at which a given participant would just begin to respond and be able to discern a CO-combination (AF2) and end to respond (AF5), because response accuracy and confidence are already perfect at the higher AF. AF1 was then defined by incrementing AF2 by 2.5 Hz in order to test an AF where a given subject would be unable to perform the task. AF3 was defined as the frequency where the participant gave the maximum number of responses. AF4 was defined as the median distance between AF5 and AF3. Note, we defined individual AFs covering the performance range in the MEG experiment based on the number of responses given at different AFs of the Staircase experiment (Fig. 1C). A given AF does not necessarily correspond with the same response accuracy level (% correct responses) in each participant.

### MEG Control experiment

The design of the control experiment closely mirrored that of the CO-binding experiment. Participants were presented with the exact same stimuli at their individual AFs (AF1-5), except that they were instructed to ignore the alternating CO-pairs, but report the number of occurrences of a dimmer center cross. A “dim” consisted of the center cross changing from its original white to grey (RGB-value: 65, 65, 65) for 350 ms, which randomly happened 1-3 times in a time range of 700 ms after alternation onset and 700 ms before alternation offset. The number of dims was counterbalanced, and their presentation order was randomized across blocks. The duration of the pause between two dims varied randomly but lasted at least 400 ms. Participants were to indicate the frequency of dims by selecting from three alternatives (1x, 2x, 3x) by pressing the index, middle, or ring finger, respectively. The control-experiment did not include a confidence query, and participants were instructed to guess if they were unsure regarding the correct response.

### Data analysis

#### MEG data preprocessing

The MEG signal was continuously recorded during the experimental session, and then subjected to Maxwell filtering (MaxFilter Software, Elekta Instrument Ab Stockholm, Sweden), which included signal space separation (SSS,^63^, maxfilter_mci_sss_ds2) for removing environmental noise from the MEG data. All subsequent analyses were based on the magnetometer data only. The MEG signal was down-sampled to 500 Hz and then cut into trial epochs ranging 0.5 s before to 4.2 s after trial onset using routines of the FieldTrip Toolbox^64^ running under MATLAB^TM^ (R2021b.). Artefact rejection (AR) was automatized by using a customized MATLAB script that iterated through each magnetometer and each epoch in each subject to identify and exclude trials containing individual peak-to-peak jumps of activity above 3pT. This guaranteed the elimination of major artifacts (e.g., eye blinks) by rejecting on average of 5–15% of trials in each participant.

### Event-related signal averaging

Each trial epoch was cut into sub-epochs (single alternations) starting at the onset and ending at the offset of an alternation epoch (c.f. Fig. 1B). For each subject and AF condition, sub-epochs were then averaged across trial epochs and the whole experiment. For the sequential epoch analysis shown in Fig. 6A–C, averages were computed for subsequent alternations within an experimental trial, with each sequential epoch containing four consecutive alternations. An analogous approach was used for the color-selective sequential epoch analysis shown in Fig. 6E, F. Note, for the epoch-based analysis a higher temporal resolution (shorter epochs) would be desirable. However, the analysis approaches the limit of event counts necessary for event averaging within a given observer. Four alternations per epoch were used to guarantee a sufficient signal-to-noise ratio, which allowed for the analysis of at least three successive epochs.

### Current source density (CSD) analysis

After event-related signal averaging of the magnetic field responses, all data were transformed into source space using a source density model (minimum norm least squares estimates) as implemented in the source localization software Curry 8.3 (Compumedics Neuroscience USA Ltd.). The MNI-cortex segmentation provided in Curry 8.4 served as source compartment, after each subject’s sensor data were co-registered with the MNI-cortex using individual anatomical landmarks (nasion, left and right pre-auricular point).

### Definition of regions of interest (ROIs) and derivation of source waves

The retinotopically defined visual cortex areas V1/V2 (EVC) and V4/VO shown in Fig. 2A were defined based on the grand-average parcellation map of the visual cortex provided in FreeSurfer (CsurfMaps1)^65^. The individual time course of source activity in EVC and V4/VO (source waves) was then derived from ROIs centered at the CSD maximum in respective regions. The source waves represent the CSD-average over the current dipoles contained in the ROI, which subtended a spherical range with a diameter of 3.7 mm and encompassed on average 6 current dipoles.

### Statistical data validation

For derivation of the range of chance performance (Figs. 2 and 4), the binomial confidence interval of chance performance was estimated (*p* = 0.05, *n* = 45, and nominal chance level of 50%) using the *binocdf* function in MATLAB R2021b, which yielded a range of 50 ± 12.5%. Statistical validation of the peak latency and amplitude differences between AF conditions was performed using the repeated measures analysis of variance (rANOVA) approach (*ranova* function in MATLAB R2021b).

### Sigmoid function fitting

For fitting the sigmoid function to A-time-minus-REC-time differences shown in Fig. 4B, we used the “curveFitter” app available in MATLAB R2024a. The fitting was performed a sigmoidal Gompertz model [val(*x*) = *d* + (*a*-*d*)*exp(-exp(-b*(*x*-*c*)))], yielding the following parameters: *a* = 96.52, *b* = 0.02761, *c* = 2.099, and *d* = 49.44 with an R-square of 0.747.

### Granger causality analysis

The Granger causality analysis (GCA, Fig. 2D) was computed using the block-wise Granger causality method^66^ (gctest function in MATLAB R2021b) with a VAR(4) model testing time lags up to 8 ms. The GCA was performed as a sample-by-sample time-sliding analysis (40 ms sliding window) using the source-activity in EVC as predictor of activity in V4/VO (feedforward analysis), or source-activity in V4/VO as predictor of activity in EVC (feedback analysis).

### Reporting summary

Further information on research design is available in the Nature Portfolio Reporting Summary linked to this article.

## Supplementary information

Reporting Summary

Transparent Peer Review file

## Source data

Source Data

## Data Availability

All data supporting the findings of this study are available within the paper. Source data are provided for this paper accessible at OSF https://doi.org/10.17605/OSF.IO/6T325. Source data are provided with this paper.

## Code availability

Custom code for transforming Curry8 to FreeSurfer data is available at https://osf.io/vt9dk.
