# Supplementary material for: Neural limits of temporal feature binding in human visual cortex
Source: Nat Commun. 2026 Oct 26;17:11115. doi: 10.1038/s41467-026-78974-z (PMC13710965; doi:10.1038/s41467-026-78974-z)
Supplement: Supplementary file 2 [file 41467_2026_78974_MOESM2_ESM.pdf]

nature portfolio

Peer Review File

Neural limits of temporal feature binding in human visual cortex

Corresponding Author: Professor Jens-Max Hopf

This file contains all reviewer reports in order by version, followed by all author rebuttals in order by version.

Version 0:

Reviewer comments:

Reviewer #1

(Remarks to the Author)
This study demonstrates a critical role for neural feedback from higher to lower levels of the visual cortex in the binding of spatially separated color and orientation features. This recurrent, early visual cortical (REC-EVC) response is elicited over the time range 100-160 ms after stimulus delivery in visual areas V1/V2. When paired color and orientation stimuli were presented at a high rate such that the REC-EVC was interrupted by evoked activity from the subsequent stimulus, correct binding could not be achieved. The authors conclude that the integrity of the REC-EVC feedback response was an essential mechanism for achieving successful binding.
This study is a technical tour-de-force that adds to the emerging evidence that recurrent feedback to early visual areas underlies key visual-perceptual functions including attention, awareness, and discrimination. The methods are sound, and the paper is well-written. This study represents an important advance in our understanding of the cortical mechanisms of visual perception. I have a few suggestions for streamlining the manuscript for the non-specialist reader and for clarifying some technical points.
For streamlining the presentation, I would suggest dropping (or simplifying) Fig. 3, since Fig. 4 delivers essentially the same information in a more digestible form. If I understand Fig. 3 correctly, the duration of the blue shaded area is the interstimulus interval for the AF1 condition, while the duration of the blue + red areas is the ISI for AF2, and the duration of the blue + red + orange areas is the ISI for AF3. It takes a while to figure this out, and if you decide to keep the figure maybe the graphics could be simplified. However, I think the non-specialist reader would be happy going from Fig. 2 to Fig. 4.
Here are some additional, mostly minor points:
--Line 61. Maybe “proposed” instead of “supposed”
--Line 85. Not sure what “seize” means. Maybe “becomes less accurate”?
--Line 113. “….all trials of the experiment, separately for each AF…”
--Line 127. Could you describe the procedure for concatenating the waveforms. How were those dashed waveforms obtained?
--Line 155. “(B) Latency range of the REC-EVC peak….”
--Lines 256-257. I don’t understand why the REC-EVC could not be subject to instantaneous attentional modulation with the first alternation. Maybe attention could be tonically applied at or before trial onset, and then become more effective as the trial continued.
--Lines 283-285. I don’t think Fig. 6B shows data from the control experiment. That would be Fig. 6C.
--Line 286. Should be 0.6-4 s, not ms?
--Line 289. Maybe should be “successive” instead of “subsequent”?
--Line 290. The (E) should actually be (F)
--Line 365. Is “FB-EVP” defined somewhere? Is it the same as REC-EVP?
--Line 370. Is “FBA” defined somewhere?
--Line 373. A couple of other studies showing feedback to V1/V2 in the latency range 150-200 ms during an attention task: T. Noesselt et al. Neuron 2002, and F. DiRusso et al., Cerebral Cortex 2003,
--Lines 407-421. Towards the end of the Discussion it would be important to re-emphasize the point made in the Introduction that the mechanism for binding spatially separated features may be quite different from the mechanism of binding features that belong to the same object. The term “feature binding” is usually applied to multi-featured objects, whereas with spatially separated items the term “pairing” may be more appropriate (as in Line 390). This type of binding is very different from the “gluing” of features that are incorporated into the same object. Indeed, is it not possible that the REC-EVC response may be a requirement for the accurate perception of any type of stimulus pair, features or not, that can achieve “stroboscopic

stationarity". So the REC-EVC may not be specific to feature binding, but represents a more general perceptual mechanism. Some discussion along these lines would be a welcome addition to the Discussion.
--Line 490. Maybe "initiated" instead of "initialized".
--Line 493. Please explain why each trial began with 0.6 s of alternations with a mask. A warm-up?
--Lines 514-521. I would suggest moving this paragraph up to follow line 500,, where AFs are first described.
--Line 517. Change "responds" to "respond"
--Line 518. Change "end to respond" to something like "no longer responds".

Reviewer #2

(Remarks to the Author)
This paper investigates MEG source-level activity of human observers in a colour-orientation binding experiment with the aim to reveal the neural mechanisms underlying feature binding in the visual domain. Previous work by Holcombe and Cavanagh (2001) has shown that feature binding across different spatial locations requires considerably more time compared with feature binding at the same spatial location or within the same object. These findings suggest that feature binding across space requires slower, re-entrant feedback processing in the visual system. Here, the authors examine the time course of early visual cortex activity at different feature alternation speeds. The results indicate that feature binding breaks down when the alternation rate exceeds the required time of the re-entrant process. They also show data from a control experiment with the same stimuli but without the task demands for feature integration, which suggests that binding is subject to top-down modulations.

This is an interesting study investigating a well-established psychophysical effect by means of MEG source analysis. The employed experimental procedures and statistics are sound, and the analytical means are elegant providing largely convincing results. Overall, the paper is clearly written, and the figures are nicely designed. Nevertheless, I do have several recommendations for improvement, about which you can read in greater detail below.

Major comments:

1. One major point is the claim for re-entrant activity from higher visual areas (V4 / VO) to drive the effects in early visual areas (V1/V2). The authors mostly base this argument on a later activity maximum in early visual activity (V1/V2). Unfortunately, the MEG activity time courses in higher-level visual areas (V4 / VO) are not shown. Consequently, it is hard to validate whether the shown effects really isolate re-entrant, feedback processes from higher to lower visual areas. I recommend showing the MEG source activity time courses also for the V4 / VO – region of interest. Moreover, it would be useful to investigate the functional connectivity profile between V1/V2 and V4/VO – areas, possibly by means of Granger-causality analysis, to better justify the existence of re-entrant activity from higher-level areas to low-level visual cortex. Given the overall rationale of the paper, this would motivate several hypotheses:

(I) One would predict a breakdown of connectivity when the alternation time exceeds the recurrent-processing time per participant

(II) One would predict less connectivity in the control experiment compared with the feature-binding experiment

2. A second major point is the claim for the influence of colour-based vs. rhythmic attention. In my eyes, this argument is somewhat trivial given that the employed task strongly biases observers to explicitly focus on one particular colour rather than on both colours in alternation. This is so, because the response on a every trial is based on which orientation was paired with the red colour, rather than randomly intermixing red and green colours. If I recall correctly, this was done differently in the original study by Holcombe and Cavanagh (2001) and possibly also in the present psychophysics experiment. Please clarify whether and if so, why you have adapted your paradigm for the MEG experiment, and whether this may have altered your results. Moreover, I would recommend a more cautious interpretation of your findings with respect to colour-based vs. rhythmic attention given this confound.

3. It is stated that the alternation frequencies were titrated individually for each participant based on their behavioural performance in the prior psychophysics experiment. If this is the case, why is there considerable between-participant variation in performance shown in Figure 3? Please, clarify this logical inconsistency.

4. In general, I would need more information about the source reconstruction (specifically the region-of-interest selection) to better evaluate its validity.

Further, there are some minor inconsistencies in the paper.

1. Please delete the "in" in the sentence "alternating in at different …" (l. 31 in the abstract).
2. The introduction example is somewhat ill-posed (ll. 50/51): "When a brake-light suddenly turns on ahead of us, we must instantly realize what car it belongs to, and how fast it approaches us."

I would argue that the car is not approaching us once the driver hits the brake. Instead, we approach the static car then.

3. Please, better describe the use of the mask in the experiment, and what it is used for, and why the masked display sequence is different from the tracking period.

4. l. 83: The colour in the figure is actually “pink”, not “purple”.
5. l. 197: I cannot see a “red circle” in Figure 4A.
6. l. 205: You probably mean “chance”, not “change”.
7. The units of the MEG activity time courses are very hard to read. Moreover, they repeatedly report “micro-Ampere”, but shouldn’t it be “micro-Tesla” given that it is MEG?
8. The notions for the main and control experiments are mixed up in the Figure 6B and C legends (ll. 283-285).
9. l. 311: There is a reference to Herrmann (2001) but this is not stated in the reference list.
10. ll. 379-403 in the discussion: You probably mean “sketch-pad”, instead of “scratch-pad”, right?

Reviewer #3

(Remarks to the Author)

Schonemann and colleagues recorded MEG activity in human participants performing a color-oriented dynamic binding task, which has previously been shown to be more demanding and associated with slower binding rates. Using time-resolved source localization, the authors identify a key neural component—the REC-EVC component—occurring around 120 ms in V1/V2. This component appears stable within individuals yet shows slight latency variation across participants, which in turn predicts individual differences in binding behavior. Furthermore, the authors provide intriguing evidence that non-spatial feature binding is guided by color-based attention, with one attended color rhythmically gating feature combinations.

Overall, this is a well-conducted study employing state-of-the-art MEG analyses to address a classically important question. The results are interesting and technically solid. I particularly appreciate the authors’ effort to probe the potential driver of feature binding—namely, selective attention to one color feature that initiates subsequent binding.

That said, I have some broader concerns regarding the theoretical contribution beyond prior literature, as well as several points that would benefit from clarification or additional analyses.

1. As acknowledged by the authors, non-spatial color-orientation binding is more demanding and slows switching rates. Could the observed effects therefore reflect increased attentional load rather than temporal feature binding per se? Although a control study without explicit binding reports showed weaker neural markers, the control task does not appear to be matched in difficulty. Clarification on this point would strengthen the interpretation.
2. Regarding Figure 2A: the authors suggest that the REC-EVC component is stable across AFs by concatenating neural responses for AF1 and AF2, which appear similar to AF3–5. However, statistical support for this claim is lacking. Figure 2B analyzes REC-EVC latency only for non-spill-over AFs (line 134). Including the concatenated AF1 and AF2 data in the statistical comparison would help substantiate the claim of consistent latency across all AFs.
3. Figure 3 requires further clarification. If red and yellow conditions each produce independent ERPs, why are only selected segments displayed rather than full ERP traces?
4. The cross-individual correlation between REC-EVC latency and binding behavior (Figure 4) is compelling. A permutation-based test (e.g., shuffling AF assignments across participants) would help confirm the robustness of this relationship.
5. For the control experiment (Figure 5A–B), which AFs were included? This should be specified.
6. Line 267, missing "in the is"
7. In Figure 6, is (B) the CO-binding experiment and (C) the control experiment?
8. I am not fully convinced by the epoch-based analysis. The division into three epochs appears somewhat arbitrary, and the larger modulation in the middle epoch raises questions about generalizability. Could the effect simply due to specific stimulus parameters?
9. Relatedly, while the finding that color-based attention rhythmically gates binding is intriguing, it remains unclear whether this reflects a general binding mechanism or a strategy encouraged by the binary task design. Would the same mechanism emerge under conditions that discourage reliance on a single feature?
10. In the legend of Figure 6, there are two panels labeled (E) and no (F).

Version 1:

Reviewer comments:

Reviewer #1

(Remarks to the Author)
The authors have made extensive revisions to the manuscript and have satisfactorily answered my critical comments and suggestions for improvement.

Reviewer #2

(Remarks to the Author)
The authors have adequately addressed my previous suggestions, coimments and concerns.

Reviewer #3

(Remarks to the Author)
The reviewers have responded to my comments and questions in a largely satisfactory manner. Although some of the more general issues remain open, I appreciate that these questions may not be fully resolvable within the scope of the current study.

Open Access This Peer Review File is licensed under a Creative Commons Attribution 4.0 International License, which permits use, sharing, adaptation, distribution and reproduction in any medium or format, as long as you give appropriate credit to the original author(s) and the source, provide a link to the Creative Commons license, and indicate if changes were made.
In cases where reviewers are anonymous, credit should be given to 'Anonymous Referee' and the source.
The images or other third party material in this Peer Review File are included in the article’s Creative Commons license, unless indicated otherwise in a credit line to the material. If material is not included in the article’s Creative Commons license and your intended use is not permitted by statutory regulation or exceeds the permitted use, you will need to obtain permission directly from the copyright holder.
To view a copy of this license, visit https://creativecommons.org/licenses/by/4.0/

Reviewer #1

This study demonstrates a critical role for neural feedback from higher to lower levels of the visual cortex in the binding of spatially separated color and orientation features. This recurrent, early visual cortical (REC-EVC) response is elicited over the time range 100-160 ms after stimulus delivery in visual areas V1/V2. When paired color and orientation stimuli were presented at a high rate such that the REC-EVC was interrupted by evoked activity from the subsequent stimulus, correct binding could not be achieved. The authors conclude that the integrity of the REC-EVC feedback response was an essential mechanism for achieving successful binding.
This study is a technical tour-de-force that adds to the emerging evidence that recurrent feedback to early visual areas underlies key visual-perceptual functions including attention, awareness, and discrimination. The methods are sound, and the paper is well-written. This study represents an important advance in our understanding of the cortical mechanisms of visual perception. I have a few suggestions for streamlining the manuscript for the non-specialist reader and for clarifying some technical points.

We thank the reviewer for this very positive evaluation.

For streamlining the presentation, I would suggest dropping (or simplifying) Fig. 3, since Fig. 4 delivers essentially the same information in a more digestible form. If I understand Fig. 3 correctly, the duration of the blue shaded area is the interstimulus interval for the AF1 condition, while the duration of the blue + red areas is the ISI for AF2, and the duration of the blue + red + orange areas is the ISI for AF3. It takes a while to figure this out, and if you decide to keep the figure maybe the graphics could be simplified. However, I think the non-specialist reader would be happy going from Fig. 2 to Fig. 4.

We agree with the reviewer that Figure 3 does not communicate the data in a sufficiently clear way. In particular, the coloration of the AF conditions is misleading (an issue also raised by reviewer #3), and it takes time to be sorted out. We followed the reviewer's suggestion to simplify the Figure 3 by (1.) changing the way the alternation times (AT) are presented. We now show non-overlapping horizontal color bars spanning from 0 to the AT which eliminates the misleading relation between the source waves and the colored time ranges. (2.) We dropped the performance diagrams on the left, but added the performance data as numbers (% correct responses) to the horizontal bars, thereby establishing a more direct link between performance at a given AF and the REC-EVC response in the source waves. Finally, we added source waves of the V4/VO ROI as suggested by reviewer #2.

--Line 61. Maybe "proposed" instead of "supposed"
--Line 85. Not sure what "seize" means. Maybe "becomes less accurate"?
--Line 113. "....all trials of the experiment, separately for each AF..."

We corrected the wording as suggested.

--Line 127. Could you describe the procedure for concatenating the waveforms. How were those dashed waveforms obtained?

We admit that the concatenation is not clearly defined in the text. We simply plotted the source-waves of AF1 and AF2 twice in a row (solid line then dashed line), such that the end of the AF continues with itself. The intention was to illustrate that the REC-EVC peak doesn't change under changing AFs but spills into the next alternation, when it does not fit anymore into the alternation time. For AF1 and AF2 the presented REC-EVC waveforms evoked by the prior alternation still exhibit the very same latency irrespective of alternation time. To overcome the misleading way of data presentation, we replaced the dashed waveforms with arrows marking the REC-EVC peak latency that is added to the AT (end of the source-wave), and revised the figure legend accordingly [Lines 231-237].

--Line 155. "(B) Latency range of the REC-EVC peak...."

We corrected the wording as suggested

--Lines 256-257. I don't understand why the REC-EVC could not be subject to instantaneous attentional modulation with the first alternation. Maybe attention could be tonically applied at or before trial onset, and then become more effective as the trial continued.

We fully agree with the reviewer that tonic top-down attention could be allocated prior to trial onset. The argument actually refers to single trial-by-trial experiments where there is no prior about the upcoming attended values. Admittedly, this does not entirely apply here, as we show that the majority of subjects, indeed, establishes an attentional bias for one or the other color throughout the whole experiment. We accordingly, changed the text and motivation of the epoch-wise analysis [Lines 321-327]. We now point out that related attention research on sustained spatial focusing has shown that attention-driven modulatory effects in visual cortex (P1 enhancement) require some trials to establish (Hillyard, Münte & Neville, 1985), leading to the hypothesis that similar gradual effects may appear in the domain of feature attention and binding.

--Lines 283-285. I don't think Fig. 6B shows data from the control experiment. That would be Fig. 6C.
--Line 286. Should be 0.6-4 s, not ms?
--Line 289. Maybe should be "successive" instead of "subsequent"?
--Line 290. The (E) should actually be (F)
--Line 365. Is "FB-EVP" defined somewhere? Is it the same as REC-EVP?
--Line 370. Is "FBA" defined somewhere?

We corrected mistakes, changed wording, and defined abbreviations as suggested.

--Line 373. A couple of other studies showing feedback to V1/V2 in the latency range 150-200 ms during an attention task: T. Noesselt et al. Neuron 2002, and F. DiRusso et al., Cerebral Cortex 2003

We thank the reviewer for suggesting this excellent literature. We have included respective citations in the Results section and at the suggested point in the Discussion section [Line 322,452].

--Lines 407-421. Towards the end of the Discussion it would be important to re-emphasize the point made in the Introduction that the mechanism for binding spatially separated features may be quite different from the mechanism of binding features that belong to the same object. The term "feature binding" is usually applied to multi-featured objects, whereas with spatially separated items the term "pairing" may be more appropriate (as in Line 390). This type of binding is very different from the "gluing" of features that are incorporated into the same object.

We have added text in the Discussion [Lines 505-507] to clarify that the color-orientation 'binding process' we refer to in this work is not of the typical within-object feature conjunction type.

Indeed, is it not possible that the REC-EVC response may be a requirement for the accurate perception of any type of stimulus pair, features or not, that can achieve "stroboscopic stationarity". So the REC-EVC may not be specific to feature binding, but represents a more general perceptual mechanism. Some discussion along these lines would be a welcome addition to the Discussion

We thank the reviewer for pointing to this important possibility. As suggested, we have extended the discussion section [Lines 497-504] to indicate that the temporal integrity of the EVC-REC process may be a more general prerequisite of visual perceptual selection processes, in particular with respect to temporal limits of backward and object-substitution masking.

--Line 490. Maybe "initiated" instead of "initialized".

We have changed wording as suggested.

--Line 493. Please explain why each trial began with 0.6 s of alternations with a mask. A warm-up?

Masking the stimulus alternation at the beginning of a trial is a very important feature of the experiment. From piloting the MEG experiment we learned that observers would often be able to discern from the very first CO-pair the correct pairing of color and orientation. To eliminate this issue, we alternated the CO-pairs with a mask for the initial 600ms of the 4 sec tracking period. We added text to the Methods section [Lines 599-602] explaining the role of the mask in more detail.

--Lines 514-521. I would suggest moving this paragraph up to follow line 500,, where AFs are first described.

We moved the paragraph as suggested.

--Line 517. Change "responds" to "respond"
--Line 518. Change "end to respond" to something like "no longer responds".

We made changes as suggested

Reviewer #2

This is an interesting study investigating a well-established psychophysical effect by means of MEG source analysis. The employed experimental procedures and statistics are sound, and the analytical means are elegant providing largely convincing results. Overall, the paper is clearly written, and the figures are nicely designed. Nevertheless, I do have several recommendations for improvement, about which you can read in greater detail below.

We thank the reviewer for the positive evaluation.

1. One major point is the claim for re-entrant activity from higher visual areas (V4 / VO) to drive the effects in early visual areas (V1/V2). The authors mostly base this argument on a later activity maximum in early visual activity (V1/V2). Unfortunately, the MEG activity time courses in higher-level visual areas (V4 / VO) are not shown. Consequently, it is hard to validate whether the shown effects really isolate re-entrant, feedback processes from higher to lower visual areas. I recommend showing the MEG source activity time courses also for the V4 / VO - region of interest. Moreover, it would be useful to investigate the functional connectivity profile between V1/V2 and V4/VO - areas, possibly by means of Granger-causality analysis, to better justify the existence of re-entrant activity from higher-level areas to low-level visual cortex. Given the overall rationale of the paper, this would motivate several hypotheses: (I) One would predict a breakdown of connectivity when the alternation time exceeds the recurrent-processing time per participant (II) One would predict less connectivity in the control experiment compared with the feature-binding experiment.

We thank the reviewer for bringing up this very important point. We entirely agree that the mere fact that V1/V2 activity follows V4/VO does not prove that the latter areas feed-back to, or modulate activity in, the former areas. We followed the reviewer's suggestion to add source-waves of the V4/VO ROI in Figure 2 and Figure 3 showing that the source activity in V4/VO displays a temporal maximum prior to V1/V2. Furthermore, we performed a Granger causality analysis as recommended showing that from approximately 100ms onwards, the Granger-causation of V4/VO->V1/V2 is significantly enhanced relative to the feedforward direction (V/V2 -> V4/VO), which supports the interpretation that the REC-EVC is subject to feed-back modulation. Respective data are now displayed as Panel D in Figure 2 and reported in the Results section [Lines 156-169], and we extended the Methods section accordingly [Lines 693-698]. We also performed an analogous analysis for AF1 where the alternation time is too short to contain the REC-EVC. This analysis yields a predominantly feed-forward but not feed-back causation within the time range of the alternation. The

results, however, require some disclaimer. Because of the 'spill-over effect in AF1, the REC-EVC response often appears very close to the stimulus onset (0ms), which renders the analysis unrepresentative. We provide the results as Figure for the reviewer's inspection here. We decided against including this analysis into the manuscript.

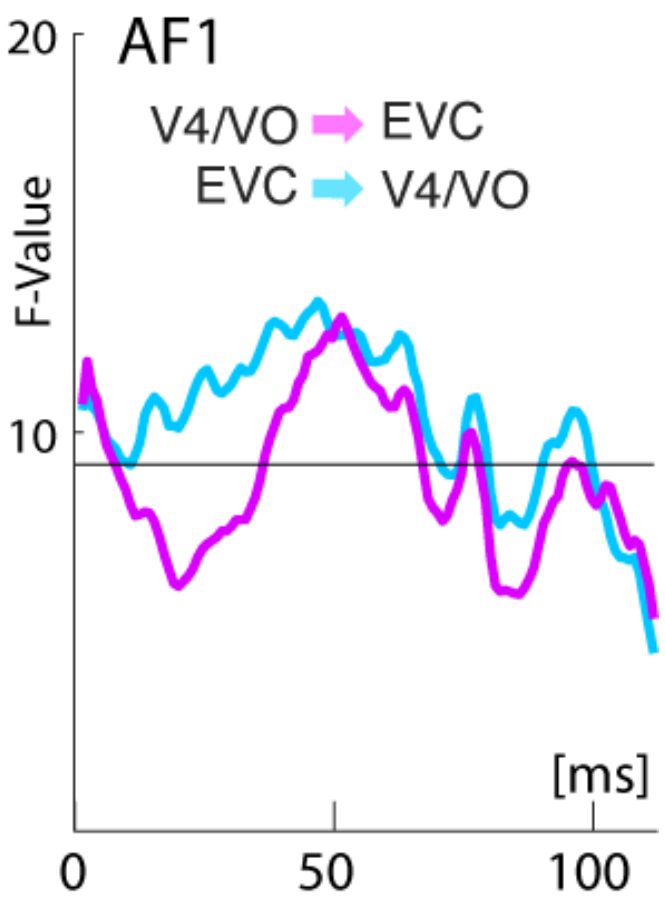

2. A second major point is the claim for the influence of colour-based vs. rhythmic attention. In my eyes, this argument is somewhat trivial given that the employed task strongly biases observers to explicitly focus on one particular colour rather than on both colours in alternation. This is so, because the response on a every trial is based on which orientation was paired with the red colour, rather than randomly intermixing red and green colours. If I recall correctly, this was done differently in the original study by Holcombe and Cavanagh (2001) and possibly also in the present psychophysics experiment. Please clarify whether and if so, why you have adapted your paradigm for the MEG experiment, and whether this may have altered your results. Moreover, I would recommend a more cautious interpretation of your findings with respect to colour-based vs. rhythmic attention given this confound.

Reviewer is right that the experimental task emphasizes attention to red as an underlying selection mechanism and that rhythmic attention as a response strategy is less likely. We would like to annotate that Holcombe & Cavanagh (2001) did in their experiments exclusively ask for the pairing of orientation with red (c.f. p.127, 2nd paragraph, last sentence). As we set out for a close replication of their psychophysical findings, we decided to use the same task instruction. Nonetheless, we entirely see the reviewer's point that a random association of color and orientation on the response screen would have been a better choice for pinpointing the attention process. In this light, it is notable that a proportion of subjects actually biased green as color, suggesting that, despite the task to report red-only pairings, participants were flexible regarding the actual color they tracked.

As to the alternative of rhythmic attention as opposed to color-based attention. We agree that rhythmic attention is less likely to underlie the REC-EVC modulation we see here. It was brought up for the simple reason that it is a conceivable mechanism and eventually less demanding than attending a specific color or orientation to stabilize one or the other percept. We have added text in the Results [Lines 376-377] and Discussion section [Lines 477-478] to clarify that the requirement to report the orientation paring with red prompted

subjects to employ to a color-based mechanism, thereby emphasizing that of rhythmic temporal attention is a possible but less likely mechanisms underlying CO-binding in the CO-binding experiment.

3. It is stated that the alternation frequencies were titrated individually for each participant based on their behavioural performance in the prior psychophysics experiment. If this is the case, why is there considerable between-participant variation in performance shown in Figure 3? Please, clarify this logical inconsistency.

We thank the reviewer for pointing to this seeming inconsistency. The AFs were not truly titrated to approach an exact response accuracy level. Instead, they were defined to cover the performance range based on the number of responses given at different AFs. While this generally reflects response accuracy, it leaves a range of variation in particular at the higher end of the AFs. For clarification, we have extended the Methods section to better explain the definition of AFs [Lines 619-622]. We also address a related issue: As we define AF1 to have subjects perform at chance, why is subject S3 performing better than chance in the MEG experiment? A likely reason is that AFs were defined based on the performance measures of the Staircase experiment which comes with specifics different from the MEG experiment. We think the major culprit is that some observers were too conservative in responding in the Staircase experiment. By nature, the Staircase must run from highest to lowest AFs. This bears the potential that subjects with conservative response settings perform actually worse than they could when tested under the conditions of the MEG experiment. Another factor is the necessity to run the Staircase experiment before the MEG experiment, so that the participants' performance may be subject to training effects playing out in the MEG experiment. We have added text in the Results section [Lines 217-222] discussing these factors potentially causing a shift in performance from the Staircase to the MEG experiment.

4. In general, I would need more information about the source reconstruction (specifically the region-of-interest selection) to better evaluate its validity.

We have extended the Methods section [Lines 671-678] by adding a paragraph reporting the details about the definition of the retinotopic areas based on Freesurfer's parcellation map of the visual cortex (CsurfMaps1). We also explained the definition of individual ROIs, from which the source-waves were derived.

Further, there are some minor inconsistencies in the paper.

1. Please delete the "in" in the sentence "alternating in at different ..." (l. 31 in the abstract).

Is corrected.

2. The introduction example is somewhat ill-posed (ll. 50/51): "When a brake-light suddenly turns on ahead of us, we must instantly realize what car it belongs to, and how fast it approaches us." I would argue that the car is not approaching us once the driver hits the brake. Instead, we approach the static car then.

We agree and changed wording to indicate that “we approach the car” [Line 53].

3. Please, better describe the use of the mask in the experiment, and what it is used for, and why the masked display sequence is different from the tracking period.

As already outlined in our response to reviewer #1, masking the stimulus alternation at the beginning of a trial turned out to be very important. From piloting the experiments, we learned that observers would often be able to discern the correct pairing of color and orientation from the very first CO-pair. To eliminate this possibility, we alternated the CO-pairs with a mask for the initial 600ms of the 4 sec tracking period. We have added text to the Methods section [Lines 599-602] explaining the role of the mask in more detail.

4. l. 83: The colour in the figure is actually “pink”, not “purple”.
5. l. 197: I cannot see a “red circle” in Figure 4A.
6. l. 205: You probably mean “chance”, not “change”.

Thanks for spotting the mistakes. We have corrected them.

7. The units of the MEG activity time courses are very hard to read. Moreover, they repeatedly report “micro-Ampere”, but shouldn’t it be “micro-Tesla” given that it is MEG?

We increased the font size of the units for better readability. The MEG waveforms we provide in the manuscript do not represent the magnetic flux measured in μT or fT, but source density estimates, which reflect current flow per area of cortex measured in μA/mm^2. Note, we corrected a mistake of the submitted version of the manuscript where we erroneously used μA/mm^3 instead of μA/mm^2 as unit of source density in the Figures.

8. The notions for the main and control experiments are mixed up in the Figure 6B and C legends (ll. 283-285).

The assignment is corrected.

9. l. 311: There is a reference to Herrmann (2001) but this is not stated in the reference list.
We added the citation to the reference list.

10. ll. 379-403 in the discussion: You probably mean “sketch-pad”, instead of “scratch-pad”, right?

Is corrected.

Reviewer #3

Overall, this is a well-conducted study employing state-of-the-art MEG analyses to address a classically important question. The results are interesting and technically solid. I particularly appreciate the authors' effort to probe the potential driver of feature binding—namely, selective attention to one color feature that initiates subsequent binding.

We thank the reviewer for the overall positive evaluation.

1. As acknowledged by the authors, non-spatial color-orientation binding is more demanding and slows switching rates. Could the observed effects therefore reflect increased attentional load rather than temporal feature binding per se? Although a control study without explicit binding reports showed weaker neural markers, the control task does not appear to be matched in difficulty. Clarification on this point would strengthen the interpretation.

The reviewer raises an important point here. In fact, we cannot rule out entirely that at least part of the modulation effects attributed to color-based/rhythmic attention reflect a higher attentional load in the color versus control task. Two observations, however, speak against attentional load accounting for the REC-EVC modulation. (1) While REC-EVC response amplitude during epoch 1 is similar between the control and CO-task, the amplitude increases and decreases over the course of the tracking period in the CO-task but not in the control task. If mere attentional load accounts for this modulation, it would be expected to appear as an amplitude increment throughout the whole tracking period. (2) The performance accuracy of the control task actually matches that of the CO-binding task. Specifically, response accuracy was very high for both, the AF5 condition of the CO-binding experiment (97.5%) and the control experiment (98.6%). A statistical comparison yielded no difference ($F(1,10)= 1.15$, $p=0.30$), rendering it unlikely that task difficulty was a major factor causing the larger REC-EVC amplitude in the CO-binding experiment.
Respective statistical comparison is now added to the Results section where we also clarify the potential contribution of attentional load to the REC-EVC enhancement [Lines 297-304].

2. Regarding Figure 2A: the authors suggest that the REC-EVC component is stable across AFs by concatenating neural responses for AF1 and AF2, which appear similar to AF3-5. However, statistical support for this claim is lacking. Figure 2B analyzes REC-EVC latency only for non-spill-over AFs (line 134). Including the concatenated AF1 and AF2 data in the statistical comparison would help substantiate the claim of consistent latency across all AFs.

We followed the reviewer's suggestion and included AF1 and AF2 into the statistical analysis, after correcting the spill-over by adding the A-time to the peak latency. Specifically, we added the range of latency variation of AF1 and 2 to the diagram in Figure 2C (red horizontal bars). We also replaced the statistical analysis of AF3-5 with an analysis testing all AFs as factor levels, and changed the Results section accordingly [Lines 145-146, 151-155].

3. Figure 3 requires further clarification. If red and yellow conditions each produce independent ERPs, why are only selected segments displayed rather than full ERP traces?

We thank the reviewer for pointing to this issue which arises from misleading coloration. The blue, red and orange range were simply meant to indicate the A-time of AF1, 2, and 3 relative to the REC-EVC peak of the AF5 condition. The problem was also raised by reviewer #1. As outlined in the response to reviewer #1, we simplified the figure by drawing the A-time as non-overlapping horizontal bars, which clarifies the issue.

4. The cross-individual correlation between REC-EVC latency and binding behavior (Figure 4) is compelling. A permutation-based test (e.g., shuffling AF assignments across participants) would help confirm the robustness of this relationship.

The reviewer points out that the latency-to-behavior relation in Figure 4 would benefit from a statistical validation. We agree with the reviewer, but would like to annotate that a permutation test using AF assignments shuffled across participants would blur but preserve the relationship, because each subject shows a decline in performance with increasing AF. We think the critical feature to validate statistically is that performance drops to change around 0ms (A-Time minus Rec-Time). We therefore fit a sigmoidal model (Gompertz function) to the data and derived the latency at the point where response accuracy enters chance performance. The very accurate fit (R-square = 0.747) yielded a latency at -6.9ms, which is very close to 0ms, thereby corroborating the conclusion that performance falls to chance almost exactly when the Rec-Time exceeds the A-Time. We have added text in the Results section [Lines 255-260], the Methods section [Lines 687-691], and changed Fig.4b to report respective results.

5. For the control experiment (Figure 5A-B), which AFs were included? This should be specified.

The control experiment all AFs were tested. Figure 5 shows the AF5 condition of the CO-binding and of the control experiment. We have added text to the figure legend for clarification [Lines 310,312].

6. Line 267, missing "in the is"

7. In Figure 6, is (B) the CO-binding experiment and (C) the control experiment?

We thank the reviewer for spotting the mistakes. We have corrected them.

8. I am not fully convinced by the epoch-based analysis. The division into three epochs appears somewhat arbitrary, and the larger modulation in the middle epoch raises questions about generalizability. Could the effect simply due to specific stimulus parameters?

For the epoch-based analysis we faced the problem that we approach the limit of event counts (in particular for the color-selective analyses) necessary for event averaging within a given observer. A higher temporal resolution (a higher number of shorter epochs) would be desirable. However, to guarantee a sufficient signal-to-noise ratio, we used 4 alternations per epoch which allowed for the analysis of three successive epochs. We have added text in the Methods section to motivate the number of epochs analyzed. [Lines 658-662]

Finally, we think the effect is unlikely reflecting specific stimulus parameters. As outlined under point #1 above, the delayed enhancement during the second epoch is a task-specific effect (absent in the control task) reflecting the requirement to perform the binding-task. The stimulus parameters of the control task were identical to those of the CO-task, except for the slight dimming of the fixation cross. If stimulus parameters defining the appearance and alternation of the CO-pairs were causing the enhancement, the enhancement should also be seen in the control experiment.

9. Relatedly, while the finding that color-based attention rhythmically gates binding is intriguing, it remains unclear whether this reflects a general binding mechanism or a strategy encouraged by the binary task design. Would the same mechanism emerge under conditions that discourage reliance on a single feature?

The reviewer raises an important point. The present experiment cannot clarify whether the rhythmical biasing of the REC-EVC process via color-based attention is specifically tied to the rigid alternation between two colors or a more general binding mechanism. A reliance on more than one feature would require to be able to establish an attentional bias for more than one feature in parallel, which is debated. We are currently running an experiment where we alternate color-color pairings. This addresses the question to some degree as it requires selecting two colors to identify the pairing. One could address the question more directly by pairing the orientation feature with a color combination, thereby requiring an attentional bias for two colors for the binding mechanism to operate. Alternatively, one could have three CO-pairings and test subjects randomly on one of the pairings. While we have no satisfying answer to the reviewer's question, we added a discussion of this point in the Discussion section [Lines 484-491], indicating that the present experiments cannot establish the generality of the mechanism, and that future experiments are needed for clarification.

10. In the legend of Figure 6, there are two panels labeled (E) and no (F).

Is corrected.
